# TGFβ1 and HGF protein secretion by esophageal squamous epithelial cells and stromal fibroblasts in oesophageal carcinogenesis

**DOI:** 10.3892/ol.2013.1409

**Published:** 2013-06-18

**Authors:** ZHIBIN XU, SHIJIE WANG, MINGLI WU, WEIWEI ZENG, XIAOLING WANG, ZHIMING DONG

**Affiliations:** 1 Departments of Endoscopy, The Fourth Hospital of Hebei Medical University, Shijiazhuang, Hebei 050011, P.R. China; 2 Pathology, The Fourth Hospital of Hebei Medical University, Shijiazhuang, Hebei 050011, P.R. China; 3 Molecular Biology, The Fourth Hospital of Hebei Medical University, Shijiazhuang, Hebei 050011, P.R. China

**Keywords:** esophageal squamous cell carcinoma, fibroblast, transforming growth factor-β1, α-smooth muscle actin, hepatocyte growth factor

## Abstract

Esophageal squamous cell carcinoma (ESCC) is an aggressive cancer with a poor prognosis. Cancer-associated fibroblasts (CAFs) affect tumorigenesis by creating an environment primed for growth and invasion through the secretion of factors, including hepatocyte growth factor (HGF) and transforming growth factor β1 (TGFβ1). In the present study, the levels of α-smooth muscle actin (α-SMA), TGFβ1 and HGF were determined immunohistochemically in oesophageal precancerous lesions (low- and high-grade intraepithelial neoplasia; LGIEN and HGIEN, respectively), carcinoma *in situ* (CIS) and squamous cell carcinoma (SCC). Immunoreactivity was observed in the cytoplasm of oesophageal epithelial cells and stromal fibroblasts. Expression levels of α-SMA, TGFβ1 and HGF increased significantly in the following order: normal, LGIEN, HGIEN, CIS and SCC. In addition, linear correlations between the expression of α-SMA, TGFβ1 and HGF and different lesions were observed. Microvessel density (MVD) was measured in all specimens and increased gradually in the normal, LGIEN, HGIEN, CIS and SCC specimens, successively. A linear correlation between MVD and pathological grade was also observed and the MVD in α-SMA-, HGF- and TGFβ1-positive groups was higher when compared with that of their negative counterparts. The results of the present study indicated that the frequent overexpression of TGFβ1 and HGF proteins, secreted by oesophageal epithelium and stromal fibroblasts, promoted the progression of oesophageal precancerous lesions via the proliferation of epithelial cells and angiogenesis, through the upregulation of vascular endothelial growth factor (VEGF) expression.

## Introduction

Oesophageal carcinoma is the fourth most common type of malignant carcinoma and has a high mortality rate in China (1). Despite long-term studies, understanding of the molecular changes underlying oesophageal carcinoma is limited. Previous studies have hypothesised that tumourigenesis represents an independent process governed by genes carried by tumour cells (2,3). However, further studies have demonstrated that carcinoma behaviour is also affected by the tumour microenvironment, including the extracellular matrix, blood vasculature, inflammatory cells and myofibroblasts (4). Notably, among these components, cancer-associated fibroblasts (CAFs) play a predominant role in carcinogenesis (5). The activation of CAFs correlates with the expression of α-smooth muscle actin (α-SMA), which is the most commonly used marker for CAFs (6,7). Numerous families of growth factors secreted by cancer cells and CAFs are involved in carcinoma initiation and progression. Transforming growth factor β (TGFβ) is capable of regulating the growth, differentiation, migration, adhesion and apoptosis of cells by binding to the TGFβ receptors (TβR-I and -II) (8). Studies have demonstrated that the TGFβ1-Smad signalling pathway is involved in the progression and prognosis of several types of carcinomas, including oesophageal (9), colorectal (10) and gastric (11) carcinoma. Hepatocyte growth factor (HGF) is a multifunctional cytokine produced by tumour cells and myofibroblasts in tumour stroma. HGF exerts multiple functions, including cell proliferation, migration and metastases (12,13), by binding to c-met, a receptor expressed in epithelial cells. The majority of previous studies have analysed TGFβ1 (9,14) and HGF (15,16) protein expression in oesophageal squamous cell carcinoma (ESCC) but not in precancerous lesions. In addition, few studies have investigated the functional and clinical significance of TGFβ1 and HGF proteins, secreted by dysplasia epithelial cells, cancer cells and stroma fibroblasts, in oesophageal carcinogenesis. Therefore, the present study aimed to examine the significance of TGFβ1 and HGF proteins in oesophageal carcinogenesis and angiogenesis.

## Materials and methods

### Tissue collection and processing

A total of 136 patients, 88 males and 48 females, treated at The Fourth Hospital of Hebei Medical University (Hebei, China) between August, 2006 and August, 2010, were enrolled in the current study (mean age, 62 years; range, 46–75 years). Normal oesophageal tissue, obtained from a distance of >5 cm between the normal oesophageal tissue and the edge of the cardiac carcinoma, was obtained from patients undergoing cardiac carcinoma resection. Oesophageal precancerous lesions were obtained from patients undergoing endoscopic mucosal resection for oesophageal precancerous lesions, and oesophageal carcinoma specimens were obtained from patients undergoing oesophagectomy surgery. Written informed consent was provided by all participants and the study was approved by the ethics committee of Hebei Medical University. Individuals had not undergone radiotherapy and chemotherapy prior to oesophagectomy or gastrectomy, and tissues were fixed with 85% alcohol, embedded with paraffin and serially sectioned at 5 *μ*m. Sections were mounted onto histostick-coated slides (Haimen Experiment Equipment Factory, Jiangsu, China) and four to five adjacent ribbons were collected for haematoxylin and eosin and immunohistochemical staining.

### Classification of pathology

In accordance with the Classification of Tumours of the Digestive System established by the World Health Organisation (17), 136 specimens were divided into 5 groups according to tissue type. These included normal, low- and high-grade intraepithelial neoplasia (LGIEN and HGIEN, respectively), carcinoma *in situ* (CIS) and squamous cell carcinoma (SCC), and comprised 20, 26, 44, 23 and 23 cases, respectively.

### Immunohistochemistry (IHC)

Paraffin-embedded sections were deparaffinised with xylene and rehydrated. Sections were incubated with H_2_O_2_ (concentration, 3%) for 30 min at room temperature. Sections were immersed in 0.01 M citrate buffer (pH 6.0) at 95°C for 10 min for antigen retrieval, and then immersed in phosphate-buffered saline (PBS) for 15 min at room temperature. Following blocking, the sections were incubated at 4°C overnight with primary antibodies, including mouse anti-human α-SMA monoclonal (1:100), mouse anti-human CD34 monoclonal (1:80; Beijing Zhongshan Jinqiao Biotechnology Co., Ltd., Beijing, China), rabbit anti-human TGFβ1 polyclonal (1:100; Bioworld Technology Inc., Nanjing, China) and rabbit anti-human HGF polyclonal (1:100; Santa Cruz Biotechnology, Inc,, Santa Cruz, CA, USA) antibodies, and subsequently washed with PBS. Sections were incubated with biotin-conjugated goat anti-mouse or rabbit IgG at 37°C for 1 h, and visualization was achieved with peroxidase-labelled streptavidin-biotin and diaminobenzidine. Slides were subsequently counterstained with Mayer’s haematoxylin, dehydrated and mounted.

### Interpretation of IHC

Cytoplasmic staining of HGF and TGFβ1 was scored by the percentage of positive cells (0, <10%; 1, 10–25%; 2, 26–50% and 3, >51%), where 0 was classified as negative expression (−) and 1–3 was classified as positive expression (+). A specimen of invasive breast and hepatic carcinoma served as a positive control for TGFβ1 and HGF, respectively. Absence of α-SMA immunostaining in the myofibroblasts was classified as negative (−) and immunostaining patterns, including focal or diffuse, weak or strong, were classified as positive (+).

Anti-CD34 antibody was used to stain endothelial cells and detect microvessel density (MVD), as described previously (18). A single endothelial cell or cluster of endothelial cells, with or without a lumen, was hypothesised to represent individual vessels. However, vessels with thick muscular walls or of a caliber of >8 red blood cells were excluded. Highly vascular areas were identified by scanning sections at low power magnification (×100) to determine three hot spots. The MVD is presented as the mean of the highest three counts at high power magnification (×200), and the slides were interpreted by two independent observers (Xiaoling Wang and Zhiming Dong).

### Statistical analysis

Results were analysed using SPSS software, version 13.0 (SPSS, Inc., Chicago, IL, USA). Comparison of the expression of α-SMA, TGFβ1 and HGF among various clinical and histological parameters was performed using the Pearson’s χ^2^ test and the Fisher’s exact test. Correlations among the various factors were analysed using the Spearman’s rank correlation and values of MVD were analysed with analysis of variance and Dunnett’s tests. Data are presented as the mean ± standard deviation. A two-sided P<0.05 was considered to indicate a statistically significant difference.

## Results

### Stromal fibroblast expression of α-SMA in oesophageal carcinogenesis

**Figure 1.**
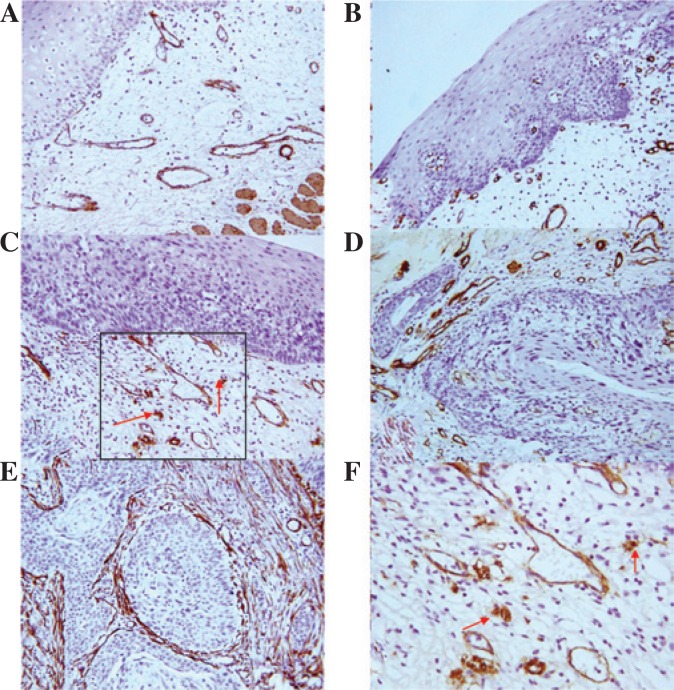
Expression of α-SMA in stromal fibroblasts in esophageal carcinogenesis. (A and B) α-SMA-negative NM and LGIEN tissues, respectively (SP staining; magnification, ×200). (C–E) α-SMA-positive expression in HGIEN, CIS and SCC tissues, respectively (SP staining; magnification, ×200). (F) Magnification of Fig. 1C (SP staining; magnification, ×400). Red arrows indicate myofibroblast staining. α-SMA, α-smooth muscle actin; NM, normal; LGIEN, low-grade intraepithelial neoplasia; HGIEN, high-grade intraepithelial neoplasia; CIS, carcinoma *in situ*; SCC, squamous cell carcinoma; SP, streptavidin-peroxidase.

The IHC results showed ascending rates of stromal fibroblast expression of α-SMA in oesophageal carcinogenesis. The majority of α-SMA-positive fibroblasts were distributed in the oesophageal stroma surrounding cancer nests or adjacent to dysplasia cells (Fig. 1). No significant differences were identified between the positive rates of α-SMA expression with respect to gender and age. The positive rates of α-SMA expression in the HGIEN, CIS and SCC groups were statistically significant when compared with that of the normal group; however, no significant difference in α-SMA expression rates was identified between the LGIEN and normal groups.

### Expression of TGFβ1 and HGF in oesophageal carcinogenesis

**Figure 2.**
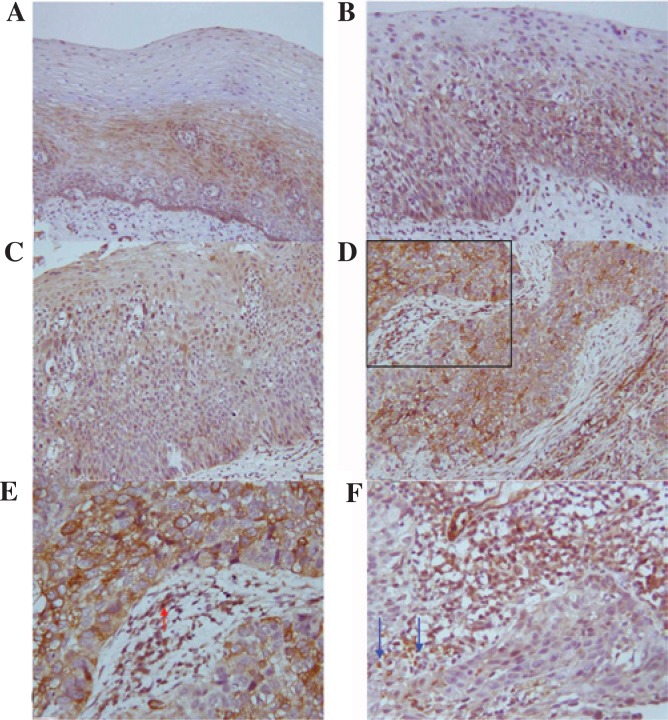
Expression of TGFβ1 in esophageal carcinogenesis. (A–D) TGFβ1-positive LGIEN, HGIEN, CIS and SCC tissues, respectively (SP staining; magnification, ×200). (E and F) TGFβ1-positive expression in myofibroblasts (red arrows) and inflammatory cells (blue arrows) (SP staining; magnification, ×400). TGFβ1, transforming growth factor β1; LGIEN, low-grade intraepithelial neoplasia; HGIEN, high-grade intraepithelial neoplasia; CIS, carcinoma *in situ*; SCC, squamous cell carcinoma; SP, streptavidin-peroxidase

**Figure 3.**
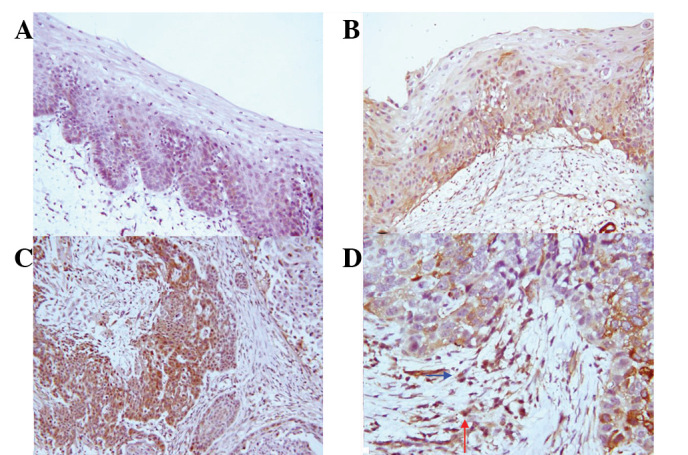
Expression of HGF in esophageal carcinogenesis. (A–C) HGF-negative LGIEN, HGIEN and SCC tissues, respectively (SP staining; magnification, ×200). (D) HGF-positive stromal myofibroblasts (red arrows) and inflammatory cells (blue arrows) (SP staining; magnification, ×400). HGF, hepatocyte growth factor; LGIEN, low-grade intraepithelial neoplasia; HGIEN, high-grade intraepithelial neoplasia; SCC, squamous cell carcinoma; SP, streptavidin-peroxidase.

The majority of TGFβ1 and HGF expression was localised in the cytoplasm of tumour and dysplasia cells, and positive cells were distributed in the proliferative basal cell zone. In the ESCC tissues, positive staining of TGFβ1 and HGF was observed in stromal fibroblasts and inflammatory cells adjacent to tumour cells, particularly at the invasive edges of tumours (Figs. 2 and 3). Ascending positive expression levels of TGFβ1 and HGF were observed in oesophageal carcinogenesis. The positive immunostaining rate for TGFβ1 and HGF was low in the normal group but increased progressively from LGIEN to HGIEN, CIS and SCC groups, successively (Table I). A significant difference in TGFβ1 and HGF expression was observed between the normal epithelia and the epithelia of LGIEN, HGIEN, CIS and SCC (with the exception of LGIEN for HGF). TGFβ1 and HGF expression exhibited a linear correlation with the progression of the various lesions (P<0.05). The values of TGFβ1 and HGF linear correlations with various lesions were −0.356 and −0.437, respectively.

No significant differences were identified between TGFβ1 and HGF expression with respect to gender and age. However, a significant difference was identified in TGFβ1 and HGF expression in ESCC and each stage of oesophageal precancerous lesions when compared with that in the normal group.

### Correlation between α-SMA and TGFβ1 expression in oesophageal carcinogenesis

The frequency of TGFβ1 overexpression was higher in α-SMA-positive groups when compared with that of the α-SMA-negative groups. The correlation between α-SMA and TGFβ1 was positive and statistically significant [correlation coefficient (r), 0.365; P=0.000; Table II].

### MVD among various clinical and histological parameters

**Figure 4.**
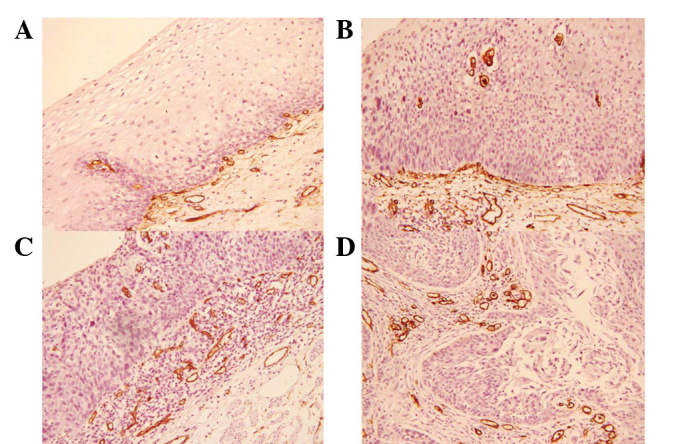
MVD in various esophageal lesions (SP staining; magnification, ×200). (A) NM, (B) HGIEN, (C) CIS and (D) invasive SCC. MVD, microvessel density; NM, normal; LGIEN, low-grade intraepithelial neoplasia; HGIEN, high-grade intraepithelial neoplasia; CIS, carcinoma *in situ*; SCC, squamous cell carcinoma; SP, streptavidin-peroxidase.

No significant differences in the MVD were identified with regard to gender and age. The MVD was 12.3±1.6, 15.7±1.9, 20.9±2.2, 21.4±1.9 and 22.0±2.3 in the normal, LGIEN, HGIEN, CIS and SCC groups, respectively. The higher values of MVD in the HGIEN, CIS and SCC groups were statistically significant when compared with that of the normal and LGIEN groups. α-SMA-, HGF- and TGFβ1-positive groups exhibited significantly higher MVDs when compared with that of their negative counterparts (Table III; Fig. 4).

## Discussion

Activated fibroblasts in tumour stroma are known as CAFs and are commonly identified by the expression of α-SMA (19,20). Studies have demonstrated that CAFs are significant promoters of tumour growth and progression via growth factors, including TGFβ1 and HGF, which are secreted by the CAFs themselves and/or by carcinoma cells (21). In the present study, positive α-SMA expression rates increased from LGIEN to HGIEN, CIS and SCC groups, successively. The majority of α-SMA was localised to atypical fibroblasts (AFs) and CAFs surrounding cancer nests. The expression pattern of α-SMA from LGIEN to HGIEN and CIS groups was weak and focal. By contrast, marked and diffuse staining of α-SMA was observed in the ESCC tissues, particularly in invasive carcinomas. Of note, overexpression of TGFβ1 positively correlated with the number of α-SMA-positive fibroblasts. Numerous studies (22–25) have demonstrated that TGFβ1 is capable of inducing α-SMA-negative fibroblasts into α-SMA-positive fibroblasts. The specific mechanism has been hypothesised to involve TGFβ1 activation of RhoA, which induces α-SMA expression via activation of the endothelial growth factor receptor (26).

Previous studies have demonstrated that overexpression of TGFβ1 (27) and HGF (28) is associated with advanced stage oesophageal (Barrett’s) adenocarcinoma. However, few studies have analysed the overexpression of TGFβ1 and HGF in oesophageal squamous cell carcinogenesis. In the present study, carcinoma cells and CAFs surrounding cancer nests markedly expressed TGFβ1 and HGF in ESCC when compared with that of precancerous lesions, particularly at the invasive edges of the carcinoma. Expression of TGFβ1 and HGF in inflammatory cells was also shown in regions adjacent to cancer nests. In addition, the positive staining rates of TGFβ1 and HGF increased significantly in the LGIEN, HGIEN, CIS and SCC groups, when compared with that in the normal group. Thus, the immunoreactivity of TGFβ1 and HGF occurred during the early stages of oesophageal carcinogenesis. No significant differences were identified between TGFβ1 and HGF expression in the HGIEN, CIS and SCC groups. However, TGFβ1 and HGF expression levels showed linear correlations with oesophageal pathological grade. It has been hypothesised that the overexpression of TGFβ1 and HGF may be involved in hyperproliferation of oesophageal epithelial cells.

Angiogenesis is an essential step for the transition of a small cluster of harmless cells into a large tumour. ESCC is a highly angiogenic tumour and biochemical studies (29) have shown that the TGFβ-vascular endothelial growth factor (VEGF) pathway may induce vascular network formation during fibroblast activation in the ESCC stroma. TGFβ1 may be associated with gastric tumour progression by indirectly stimulating angiogenesis through the upregulation of VEGF expression (30). In addition, HGF is a significant angiogenic growth factor involved in the progression of ESCC (31,32). In the current study, the MVD increased rapidly through the normal, LGIEN, HGIEN, CIS and SCC groups, successively. However, no significant differences were identified in the MVD between the HGIEN, CIS and SCC groups. Coexpression of TGFβ1 and HGF was examined, and the correlations with MVD were evaluated in the 136 specimens. The values of MVD in the LGIEN, HGIEN, CIS and SCC groups with positive TGFβ1 and HGF expression were higher compared with that in the groups that were negative for TGFβ1 and HGF (data not shown). The results of the present study have indicated that TGFβ1 and HGF contribute to oesophageal angiogenesis at the stage of initiation via their respective pathways.

From the results of the present study and previous studies performed in mouse models (33,34), we hypothesise that the suitable microenvironment created by AFs and CAFs in the stroma not only contributes to cancer progression but also to angiogenesis in oesophageal precancerous lesions and carcinoma. In addition, TGFβ1 and HGF may be important for oesophageal carcinogenesis and angiogenesis.

## Figures and Tables

**Table I.** Expression of α-SMA, TGFβ1 and HGF at various clinical and histological parameters.

Characteristic	n	α-SMA		TGFβ1		HGF	
		
		n (%)	P-value	n (%)	P-value	n (%)	P-value
Gender							
Male	88	40 (45.5)		46 (52.3)		28 (31.8)	
Female	48	19 (39.6)	0.509	32 (66.7)	0.105	8 (16.7)	0.056
Age, years							
<60	61	26 (42.6)		36 (59.0)		16 (26.2)	
≥60	75	33 (42.7)	0.872	42 (56.0)	0.724	20 (26.7)	0.954
Histological type							
NM	20	0 (0.0)		1 (5.0)		0 (0.0)	
LGIEN	26	3 (11.5)	0.246^a^	15 (57.7)	0.000^a^	1 (3.8)	0.375^a^
HGIEN	44	18 (40.9)	0.001^a^	29 (65.9)	0.000^a^	13 (29.5)	0.006^a^
CIS	23	15 (65.2)	0.000^a^	16 (69.6)	0.000^a^	9 (39.1)	0.002^a^
SCC	23	23 (100.0)	0.000^a^	17 (70.0)	0.000^a^	13 (56.5)	0.000^a^

a Compared with NM. α-SMA, α-smooth muscle actin; TGFβ1, transforming growth factor β1; HGF, hepatocyte growth factor; NM, normal; LGIEN, low-grade intraepithelial neoplasia; HGIEN, high-grade intraepithelial neoplasia; CIS, carcinoma *in situ*; SCC, squamous cell carcinoma.

**Table II.** Correlation between α-SMA and TGFβ1 protein expression.

α-SMA	TGFβ1		r	P-value

	−	+		
−	45	32		
+	13	46	0.365	0.000^a^

a P<0.05, vs. correlation coefficient. α-SMA, α-smooth muscle actin; TGFβ1, transforming growth factor β1.

**Table III.** MVD at various clinical and histological parameters.

Characteristic	n	MVD	P-value
Gender			
Male	88	21.2±3.5	
Female	48	20.8±3.8	0.755
Age, years			
≥60	75	21.2±3.4	
<60	61	22.0±3.1	0.886
Pathological grade			
Normal	20	12.3±1.6	
LGIEN	26	15.7±1.9^a^	0.041^a^
HGIEN	44	20.9±2.2^a,b^	0.012^a^
CIS	23	21.4±1.9^a,b,c^	0.009^a^
SCC	23	22.0±2.3^a,b,c^	0.005^a^
α-SMA			
−	77	15.3±7.3	
+	59	22.8±5.6	0.044
TGFβ1			
−	58	15.6±4.9	
+	78	20.9±4.6	0.047
HGF			
−	100	15.2±3.3	
+	36	28.3±5.8	0.008

a P<0.05, vs. NM;

b P<0.05 vs. LGIEN;

c P<0.05 vs. HGIEN. MVD, microvessel density; NM, normal; LGIEN, low-grade intraepithelial neoplasia; HGIEN, high-grade intraepithelial neoplasia; CIS, carcinoma *in situ*; SCC, squamous cell carcinoma; α-SMA, α-smooth muscle actin; TGFβ1, transforming growth factor β1; HGF, hepatocyte growth factor.
